# Sub-lethal dose exposure of cyflufenamid, flutianil, quinoxyfen, trifloxystrobin and metrafenone effects on conidial germination and gene expression in the grape powdery mildew pathogen

**DOI:** 10.3389/fmicb.2026.1944688

**Published:** 2026-10-12

**Authors:** Nancy Sharma, Mohit Mahey, Rachel P. Naegele, Timothy D. Miles

**Affiliations:** 1 Department of Plant Biology, Rutgers University, New Brunswick, NJ, United States; 2 Department of Plant, Soil and Microbial Sciences, Michigan State University, East Lansing, MI, United States

**Keywords:** *Erysiphe necator*, RNA-seq, site-specific fungicides, transcriptomics, Vitis

## Abstract

*Erysiphe necator* is a destructive obligate plant pathogen that infects grapevines worldwide. Recently, specialized fungicides have been widely employed to control grape powdery mildew. However, for some fungicides their modes of action and the transcriptome changes caused in *E. necator* upon application remain unclear. In this study, a conidial germination assay was conducted on *E. necator* conidia to determine if cyflufenamid (FRAC U06), flutianil (FRAC U13), quinoxyfen (FRAC 13), trifloxystrobin (FRAC 11) and metrafenone (FRAC 50) inhibit conidial germination. Further a transcriptomic analyses of *E. necator* conidia treated with sub-lethal doses of flutianil, quinoxyfen, and trifloxystrobin were conducted at 3 and 6 h. The results showed that cyflufenamid and metrafenone did not consistently inhibit conidial germination, even at chemical concentrations as high as 100 ppm. In contrast, flutianil, trifloxystrobin and quinoxyfen effectively suppressed conidial germination. The differential gene expression analysis revealed limited overlap in differentially expressed genes compared to the control group at their respective time frames, though all fungicide applications resulted in upregulated genes involved in cellular respiration. Quinoxyfen exposure led to reduced expression of protein kinase genes, suggesting a disruption in signal transduction, while flutianil treatment showed increased expression of sugar transporters and chitinases, possibly reflecting enhanced energy demand and cell wall remodeling. Additionally, in samples treated with quinoxyfen and flutianil, there was an overexpression of major facilitator superfamily (MFS) and adenosine triphosphate-binding cassette (ABC) multidrug transporters. Overall, these findings highlight that while flutianil, trifloxystrobin and quinoxyfen effectively inhibit conidial germination, their mode of action may also trigger altered gene expression of various gene families. This knowledge is valuable for developing improved fungicide resistance management strategies for managing *E. necator*.

## 1 Introduction

Grapevine powdery mildew (GPM), caused by an obligate biotrophic fungus *Erysiphe necator* Schwein (syn. *Uncinula necator*), is one of the most destructive diseases of viticulture worldwide. This disease causes major reductions in both grape production and fruit quality (Gadoury et al., 2012). Effective control of grape powdery mildew requires multiple repetitive fungicide applications, contributing to more than 30% of the overall production expenses (Baudoin et al., 2008; Dufour et al., 2011; Fuller et al., 2014; Sambucci et al., 2019; Sharma et al., 2023). The most used fungicides include contact fungicides like sulfur, as well as systemic fungicides such as demethylation inhibitors (DMIs; FRAC 3), quinone outside inhibitors (QoIs; FRAC 11), and succinate dehydrogenase inhibitors (SDHIs; FRAC 7), all of which are applied consistently throughout the growing season (Sharma et al., 2023; Oliver et al., 2024). However, most of these fungicides have reports of fungicide resistance accumulation across the United States and Europe (Gubler et al., 1996; Wong and Wilcox, 2002; Kunova et al., 2021; Stergiopoulos et al., 2022).

In recent years, new fungicide classes, including phenyl-acetamides (FRAC U06), cyano-methylenethiazolidines (FRAC U13), aza-naphthalenes (FRAC 13), and aryl-phenyl ketones (FRAC 50), have been introduced as innovative solutions targeting powdery mildew in cereals and specialty crops. Specifically, the active ingredients cyflufenamid (FRAC U06; available since 2012), flutianil (FRAC U13; available since 2017), quinoxyfen (FRAC 13; available since 2003), and metrafenone (FRAC 50; available since 2010) have been registered for management of *E. necator*. Cyflufenamid and metrafenone are known for possessing both protectant and curative properties against *E. necator* (Lewis et al., 2016). Moreover, flutianil is highly valued for its residual, translaminar, and curative effects against powdery mildew pathogens (Kimura et al., 2020). Additionally, quinoxyfen provides protective properties with systemic movement and translocation within the plant with redistribution through vapors (Lewis et al., 2016). Although being single mode of action, these specialty fungicides are seen as a valuable resource when considering resistance risk management strategies for grape powdery mildew.

Repetitive use of fungicides with single-site mode of action has potential for risk of fungicide resistance evolution due to selection pressure. Quinoxyfen-resistant *E. necator* isolates have been detected with EC_50_ values exceeding 100 mg/L in Europe (Genet and Jaworska, 2009). Reduced quinoxyfen efficacy against *E. necator* was observed in field trials in New York in 2010 and 2011. Additionally, in a single vineyard of West Virginia, field isolates of *E. necator* have shown high levels of resistance to quinoxyfen in leaf disc assays (Feng et al., 2018). Similarly, resistance to metrafenone in *E. necator* also started appearing a few years after the introduction of the fungicide (Kunova et al., 2016) and isolates with moderate and high resistance have been detected in various European countries (Graf, 2017). Interestingly, highly resistant strains were able to thrive and sporulate at concentrations as high as 1,250 mg/L (Kunova et al., 2016). Fortunately, resistance to cyflufenamid and flutianil in *E. necator* has not yet been reported.

Although the exact mode of action of quinoxyfen is not known, initial studies showed that quinoxyfen hampers the detection of host signals that are essential for proper germling development, and potentially affects serine esterase function, causing subsequent disruptions in cellular signaling pathways (Lee et al., 2008; Wheeler et al., 2003). Unfortunately, the mechanisms of cyflufenamid, flutianil, and metrafenone are still unknown. However, to understand the underlying risk of resistance in *E. necator*, it is important to understand the mechanism of action of these specialty fungicides. Understanding the biochemical target of the fungicide is crucial to understand the relationship between target structure and fungicide activity, which can help improve and develop novel fungicides (Cools and Hammond-Kosack, 2013). Moreover, it can help identify potential resistance mechanisms and can aid in development of rapid molecular tools for detection of target-site-based fungicide resistance. Recently, a complete chromosome-scale genome of *E. necator* isolate EnFRAME01 has been constructed (Zaccaron et al., 2023). This high-quality assembly along with *in-vitro* phenotyping such as conidial germination assays can help in understanding the metabolic and physiological consequences of fungicide on the pathogen (Cools and Hammond-Kosack, 2013). Trifloxystrobin, a QoI fungicide, was used as a positive control in this study, because of its well-characterized mode of action in plant pathogens. QoI fungicides are known to disrupt mitochondrial function by binding to the Q_0_ site (ubiquinol oxidation center) on the cytochrome b (*Cytb*) gene, blocking electron flow from *Cytb* to cytochrome *c*_1_ and thereby inhibiting adenosine triphosphate (ATP) synthesis (Bartlett et al., 2002; Kunova et al., 2021). Therefore, the goal of this study was to (i) conduct conidial germination assays of *E. necator* to determine EC_50_ concentrations of cyflufenamid (FRAC U06), flutianil (FRAC U13), quinoxyfen (FRAC 13) and metrafenone (FRAC 50); and (ii) conduct transcriptomic analysis comparing trifloxystrobin, quinoxyfen and flutianil to identify common candidate genes and genomic pathways among these fungicides that could be associated with the mechanism of flutianil.

## 2 Materials and methods

### 2.1 Culturing and maintenance of *Erysiphe necator* isolate

The *E. necator* isolate EnFRAME01 was collected from grapevines cultivated in a greenhouse located in Corvallis, Oregon, US in 2018. Fresh, disease-free grapevine leaves used for culturing *E. necator* were sourced from the third-fourth node of healthy grapevines (“Chardonnay”, *Vitis vinifera*) grown in hydroponics. Before use, the leaves were treated in a 0.6% sodium hypochlorite solution, agitated for 1 min manually, and rinsed multiple times with sterile water. The EnFRAME01 isolate was grown by carefully transferring individual conidial chains onto sterilized leaves with an ethanol-sterilized eyelash. These leaves were placed in a double-Petri dish isolation setup and incubated at 21 °C under a 16-h light and 8-h darkness cycle. Every 2 weeks, conidia (EnFRAME01) were moved onto newly disinfected, healthy leaves following the same procedure using clean paintbrushes. Non-inoculated sterilized leaves were used as a control.

### 2.2 Fungicide preparation

Five fungicides were utilized in this study, all of which were commercially available products. These included Torino (10% cyflufenamid, Gowan, Yuma, AZ), Quintec (22.58% quinoxyfen, Dow AgroSciences, Indianapolis, IN), Vivando (25.20% metrafenone, BASF Corporation, Research Triangle Park, NC), Flint Extra (42.6% trifloxystrobin, Bayer, St. Louis, MO), and Gatten (4.7% flutianil, Nichino America, Wilmington, DE). Stock solutions at 1,000 ppm were prepared by dissolving each commercial fungicide in sterile distilled water, followed by the preparation of a 100-ppm working solution for each. All stock solutions were kept in the dark at 4 °C.

### 2.3 Determination of EC_50_ for each fungicide

Conidial germination was evaluated to determine the EC_50_ (ppm) values for five fungicides: cyflufenamid, flutianil, metrafenone, trifloxystrobin, and quinoxyfen. Cyflufenamid, flutianil, metrafenone, and quinoxyfen were tested using half-strength water agar, while trifloxystrobin was assessed on half-strength water agar with the addition of salicylhydroxamic acid (SHAM), an inhibitor of alternative oxidase, at a concentration of 100 μg/ml (Mondal et al., 2005). Following autoclaving, the media were cooled to 50 °C in a water bath before the fungicide was incorporated. All fungicides were tested at 0.0001, 0.001, 0.01, 0.1, 1, 10, 50, and 100 ppm concentrations. Three milliliters of each fungicide-containing medium were dispensed into individual wells of a 24-well cell plate and used on the same day for phenotyping. Similarly, fungicide-free half-strength water agar was added as a control to verify the fungicidal effect during the experiment. All wells containing fungicide treatments were randomized using a randomized complete block design.

Before use, the 24-well plates were left to air-dry in a biosafety cabinet. Conidia from 10 to 12 day-old cultures were dispersed onto these plates using an ethanol-sterilized settling tower. The fungicide-treated plate was positioned at the bottom, while a suspension dish containing the powdery mildew-covered leaf was placed above. To dislodge the conidia, a quick burst of air from an airbrush set to 25 psi was directed at the leaf for 1 s. The conidia were left undisturbed for 5 min to allow them to settle, and the evenness of distribution was confirmed using a compound microscope. Afterward, the plates were incubated in the dark at room temperature for 16 h. Upon completion of incubation, 25 conidia per well per treatment were examined under a compound microscope ( × 10 magnification) to assess germination and the length of the germ tube. Each concentration had three technical replicates and the experiment was repeated twice.

A conidium was considered germinated if the length of its germ tube exceeded its own diameter. The effective concentration (ppm) of the fungicide that caused a 50% reduction in conidial germination (EC_50_) was calculated using linear regression analysis (*y* = *m* · log_10_(*x*) + *b*) in Microsoft Excel (Microsoft, WA, USA), based on the logarithmic scale of the active ingredient concentration and the corresponding germination percentages of *E. necator* conidia (Miles et al., 2012). Fungicides that demonstrated consistent conidial germination inhibition were used for transcriptomics analysis experiments.

### 2.4 RNA extraction and sequencing

The EC_50_ concentrations for flutianil, quinoxyfen, and trifloxystrobin, as determined above, were used for the transcriptomics experiment. Fungicide-amended half-strength water agar was prepared in petri dishes for flutianil and quinoxyfen, while trifloxystrobin media also included 100 μg/ml of salicylhydroxamic acid. An autoclaved piece of cellophane was placed on top of the fungicide-amended media, and the plates were left to air-dry in a biosafety cabinet for 1 h. Once dried, conidia collected from three powdery mildew-infected leaves were dusted onto each petri dish containing fungicide-amended media using sterilized paintbrushes. The petri dishes were then incubated in the dark at 24 °C. Conidia were harvested at 3 and 6 h using sterilized paintbrushes and transferred to 1.5 ml Eppendorf tubes containing DNA-RNA shield (Zymo Research, Irvine, CA). A control experiment was performed on fungicide-free media, with conidia collected at 0, 3, and 6-h intervals. Each treatment had three biological replicates. These samples were kept at −80 °C until further processing. The timepoint 6 h was selected as *E. necator* start developing its apical appressorium for penetration at this time point (Zhang et al., 2005), while 3 h was selected as an intermediate time point, to understand the changes in the gene expression patterns in the course of action of fungicide.

For processing, samples were thawed at room temperature. Conidia were disrupted in the TissueLyser II for 1.5 min (Qiagen, Hilden, Germany). Subsequently, lysis buffer from *Quick*-RNA Microprep Kit (Zymo Research, Irvine, CA) was immediately added and samples were kept at 75 °C for 5 min. RNA extraction was then carried out manually according to the manufacturer's instructions. RNA quality was verified with HS RNA ScreenTape assay using Agilent 4200 TapeStation (Agilent, Santa Clara, CA) at Michigan State University's Research Technology and Support Facility.

The RNA samples were sent to the RTSF Genomics Core at Michigan State University for library preparation and sequencing. Libraries were prepared using the Roche KAPA mRNA HyperPrep Kit with KAPA Unique Dual-Index primers following the manufacturer's recommendations, and sequencing was conducted using Illumina NovaSeq 6000.

### 2.5 Bioinformatics analysis

The raw sequenced RNA reads are deposited in BioProject: PRJNA1474447. The 150 bp Illumina raw reads were filtered for low-quality reads, adapters were trimmed using fastp v 0.23.2 (Chen et al., 2018), with default parameters, along with “–trim-poly-g” flag to remove polyG tail. Filtered reads were aligned to the *E. necator* reference genome (GCA_024703715.1, EnFRAME01) using HISAT2 v2.1.0 (Kim et al., 2019). SAMtools v1.16.1 (Danecek et al., 2021) was used to convert SAMs to BAMs followed by sorting and indexing of bam files. FeatureCounts v2.0.6 (Liao et al., 2014) was used for extracting raw counts assigned to genes. FastQC v0.11.7 (Andrews, 2010) was used to analyze quality parameters of the raw and filtered reads, along with MultiQC v1.14 (Ewels et al., 2016) for summarizing all quality scores.

Raw counts were corrected for batch effects using Combat-Seq tool. Batch effect is described as the systematic differences across conditions and is unrelated to the biological variable in a study, in our case it is RNA collection on different days (Zhang et al., 2020). The adjusted counts matrix produced after batch effect correction was utilized for downstream analysis. The principal component analysis was performed on the normalized counts using DeSeq2 v1.44.0 (Love et al., 2014) and visualized using ggplot2 v3.5.2 (Wickham, 2016). The differential gene expression analysis involved contrasting each treatment at various time points. Comparisons were made between the control at 3 h and 6 h and the corresponding treatments at the same time points using EdgeR v4.2.1 (Robinson et al., 2010). Genes were retained if they had counts per million (CPM) ≥ (min.count/median library size × 10^6^) in at least the minimum group sample size (or the adjusted proportion for large groups) and a total read count ≥ min.total.count across all samples (Chen et al., 2016). The criteria for up- or down-regulated genes included log-fold change of 1, and an adjusted *p-value* of less than 0.05. The control treatment at 3 h and 6 h was used as baseline for comparison among treatments. Venn diagrams were made for intersecting DEGs using ggvenn v0.1.19 and patchwork v.1.3.2. All the analysis was done in R v4.4.0 (R Core Team, 2024). All the scripts are in public GitHub repo: https://github.com/mohitmahey/RNA_seq_analysis.

## 3 Results

### 3.1 *In vitro* determination of EC_50_ concentrations for all fungicides

The EC_50_ concentrations of *E. necator* for five fungicides including trifloxystrobin, quinoxyfen, metrafenone, cyflufenamid and flutianil were tested using conidial germination experiment on the fungicide-amended media. Average EC_50_ concentrations of trifloxystrobin, flutianil, and quinoxyfen were determined as approximately 0.002 ppm, 5 ppm, and 0.0001 ppm, respectively (Figures 1A–C). Interestingly, varying fungicidal concentrations of metrafenone and cyflufenamid did not have a consistent inhibitory effect on conidial germination. The relative germination inhibition of metrafenone and cyflufenamid at 100 ppm was 90% and 67.7% and at 10 ppm was 90% and 68%, respectively. The results were inconsistent across biological replicates, and most conidia exhibited at least some degree of germ tube formation. A clear dose-dependent trend was absent for both fungicides, and relative conidial germination was not reduced by 50% even at 100 ppm (Supplementary Figures 1A, B). The inconsistency can be due to the technical limitations associated with this assay system or related to the difference in the target site for these fungicides. Therefore, EC_50_ concentrations for metrafenone and cyflufenamid were not determined. Hence, the fungicides trifloxystrobin, quinoxyfen, and flutianil were selected for transcriptomics experiments, using their respective approximate EC_50_ concentrations.

**Figure 1:**
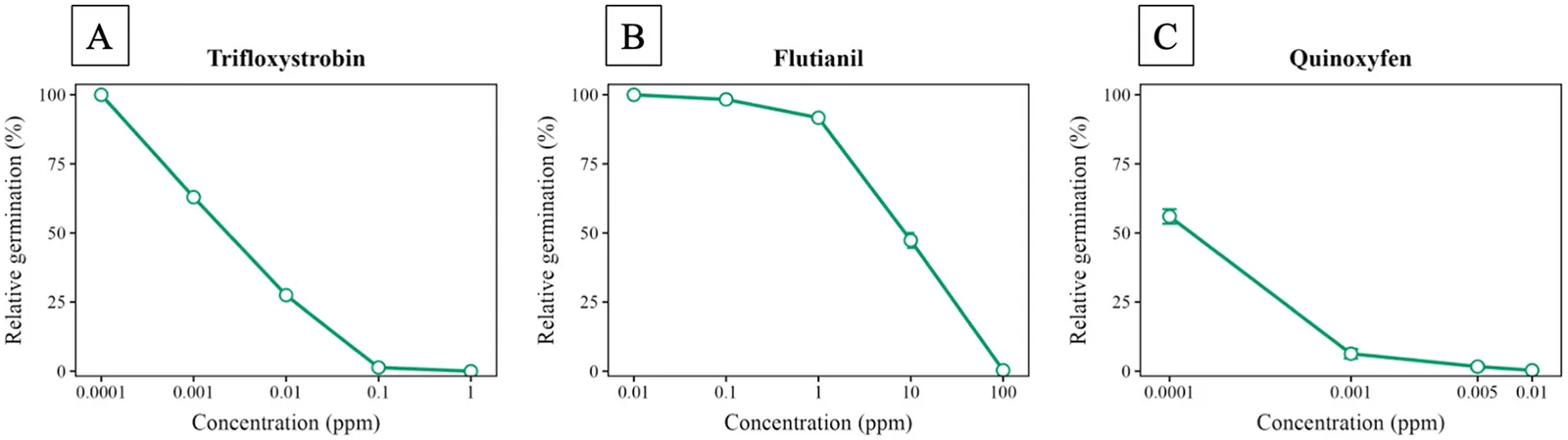
Percentage of conidial germination of *Erysiphe necator* relative to the control after treatment with fungicides at different concentrations. Conidial germination assay was conducted using a sensitive isolate of *E. necator*. Each concentration had three technical replicates and the experiment was repeated twice. Germination assay treated with **(A)** trifloxystrobin, **(B)** flutianil, **(C)** quinoxyfen. The points represent average relative germination (%) while error bars represent standard error.

### 3.2 Gene expression analysis

#### 3.2.1 RNA quality control, filtering, and metrics

The percentage of adapters trimmed averaged 11% across all samples. An average of 85.8 million reads were obtained from each sampled isolate after filtering by quality with 99% of reads passing the quality filter. The duplication rate was an average of 37% across the sampled isolates. On average, 95.69% of the reads were successfully mapped to the genome, with 87.72% mapping uniquely and 7.97% aligning to multiple locations (Supplementary Table 1). A total of 102 low-counts genes were removed, with 7,044 genes of the 7,146 genes present in the reference genome kept for differential gene expression.

#### 3.2.2 Principal component analysis

The PCA analysis summarized the variance in the transcriptomic data after applying batch correction with the combat method. The PC1 and PC2 explained 41.28% and 27.99% of the variance of data (Figure 2). The control samples at 0 time grouped near the origin or at one extreme along the PC1 axis. All fungicide-treated and control samples collected at 3 h and 6 h are clustered at the opposite end of the PCA plot, showing clear separation in gene expression profiles from the control samples taken at 0 h. The separation of 3 h and 6 h from samples at 0 h reflects developmental or incubation-associated transcriptional changes during conidial germination. Trifloxystrobin-treated samples at 3 h exhibit grouping within treatment and a clear separation from the control samples at the same time point. Moderate separation was observed by 6 h. In contrast, quinoxyfen-treated samples showed grouping within treatment, while exhibiting divergence from the control samples at 3 h, with this separation becoming even greater at 6 h. Flutianil-treated samples form a distinct cluster and are moderately separated from the control samples at both 3 and 6 h. Interestingly, flutianil-treated samples cluster relatively close to the quinoxyfen-treated samples at 3 h.

**Figure 2:**
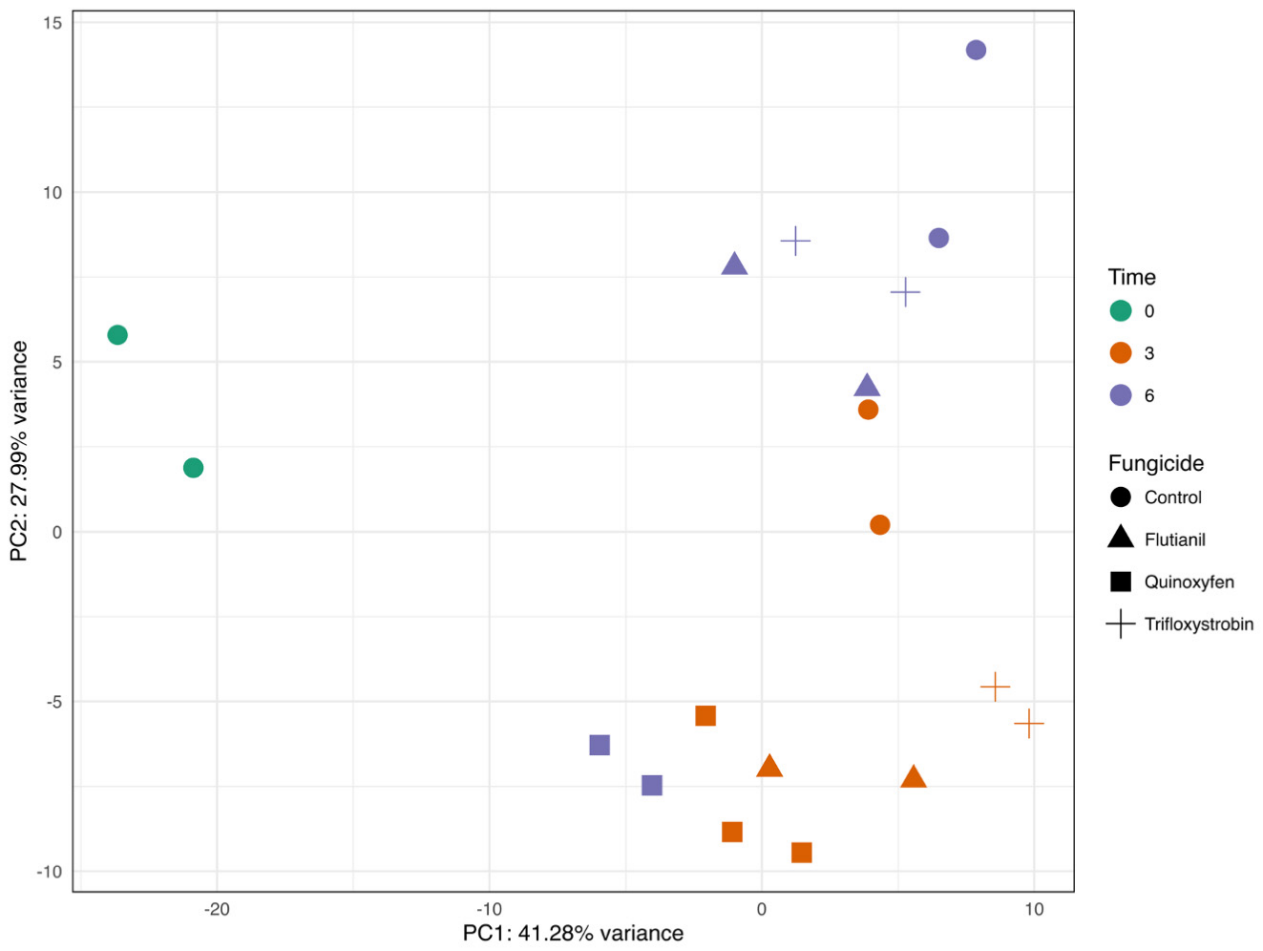
Principal component analysis of Principal component analysis of normalized counts data. The shape and color represent the fungicide treatment (quinoxyfen, flutianil, and trifloxystrobin)/control, and exposure time duration, respectively. PC1 explains 41.28% of the variance and PC2 explains 27.99% of the variance.

### 3.3 Differential gene expression at 3- and 6-h durations in quinoxyfen, trifloxystrobin and flutianil treatment

#### 3.3.1 Quinoxyfen treated change in gene expression

Gene expressions in samples treated with quinoxyfen were compared to control samples at both 3 and 6 h. This comparison aimed to identify specific expression patterns that could be related to the effects on the germination of *E. necator* conidia due to exposure to an EC_50_ dose of quinoxyfen. The DEGs of quinoxyfen-treated samples between 3 and 6 h had a huge shift with more DEGs at 6 h time points. Results indicated that, in up-regulated genes, there were 23 shared between 3 and 6 h whereas there were 36 shared down-regulated genes. Unique to 3-h timepoint, there was up-regulation of Sexual development regulator *RYP2*, putative inorganic phosphate transporter and Secreted *RXLR* effector gene. At both 3 and 6 h, velvet complex subunit 2, diphosphoinositol polyphosphate phosphohydrolase *APS1*, Oleate hydroxylase *FAH12* and Cytochrome P450 were consistently upregulated. Unique to the 6-h time point, notable upregulated genes included multiple (6) heat shock proteins, a serine protein kinase, cutinase and metabolic enzymes like NADP-dependent malic enzyme and three cytochrome P450s. Important transport proteins, such as adenosine triphosphate-binding cassette (ABC) multidrug transporter, and maltose permease or major facilitator superfamily (MFS) were also upregulated (Figure 3A and Supplementary Table 2).

**Figure 3:**
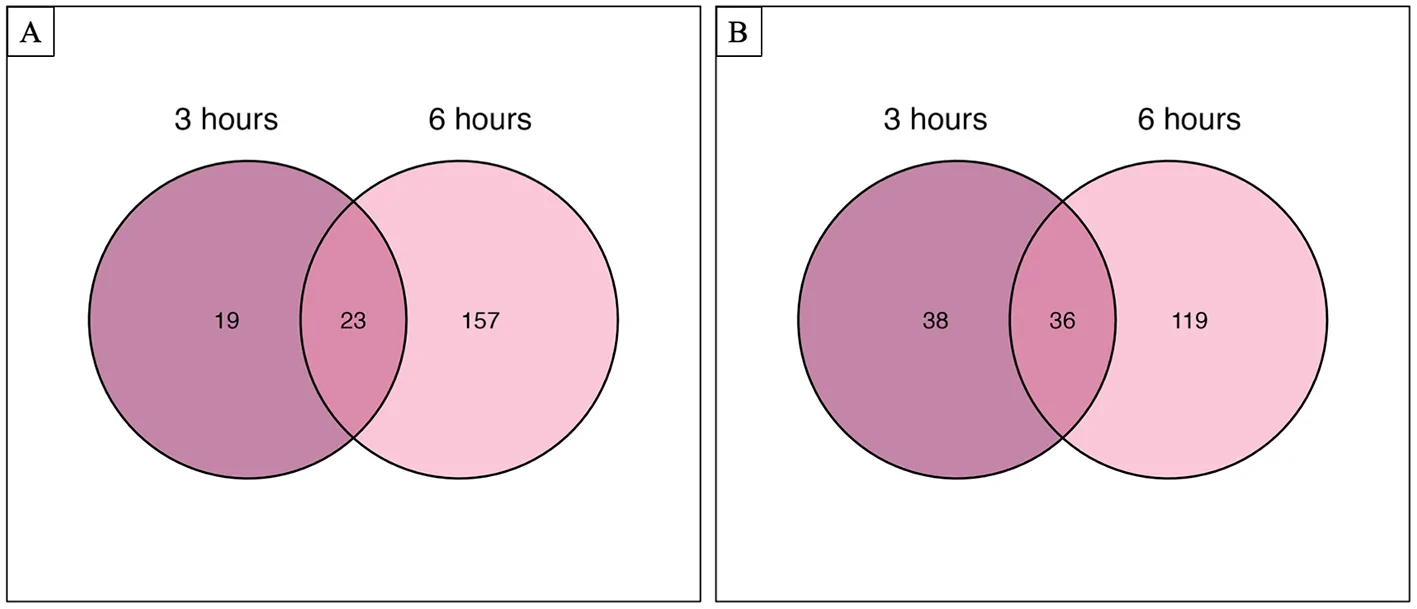
Differentially expressed genes in quinoxyfen treatment comparing 3-h and 6-h time points. **(A)** Number of up-regulated genes, and **(B)** number of down-regulated genes.

Among the down-regulated genes, a similar pattern was observed with a lot more down-regulated genes at 6-h time point as compared to 3 h. Genes that could be involved in regulatory and cellular signaling such as the transcription factor sge1 and serine/threonine-protein kinase also had reduced expression. Downregulation of ras guanine nucleotide exchange factor A (Ras-GEF A) protein was also found. Structural genes, including G2/mitotic-specific cyclin-4 and cell wall mannoprotein *CIS3* indicated impacts on cell division and structural integrity. Consistently down-regulated genes included various genes encoding transport proteins, including glutathione transporter and plasma membrane ATPase (Figure 3B and Supplementary Table 3).

#### 3.3.2 Differential gene expression in trifloxystrobin-treated samples

Gene expression was analyzed in samples exposed to an EC_50_ dose of trifloxystrobin, comparing with controls at both 3 and 6 h. Comparing the up-regulated genes at 3 and 6 h, results indicated 12 genes consistently up-regulated between time-points, while there were unique transcripts at each time point as well. NADP-dependent oxidoreductase *RED1* and maltose permease *MAL31* belonging to MFS transporters were consistently up-regulated. Interestingly, two cytochrome P450s were up-regulated at 3 h time-point but not at 6 h (Figure 4A).

**Figure 4:**
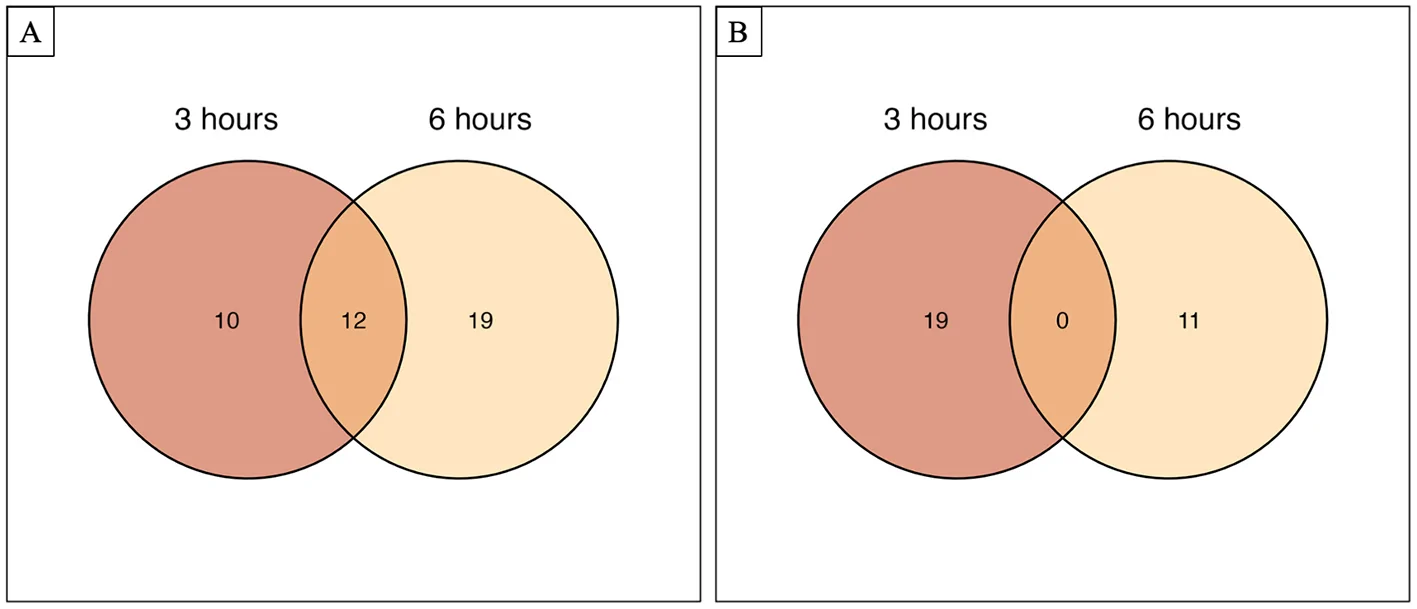
Differentially expressed genes in trifloxystrobin treatment comparing 3-h and 6-h time points. **(A)** Number of up-regulated genes, and **(B)** number of down-regulated genes.

Among the down-regulated genes, there were no shared downregulated genes at 3-h and 6-h time-points. At 3 h, the down-regulated genes included O-methyltransferase VdtC, vacuolar membrane amino acid uptake transporter *FNX2*, and potassium transport protein 1, with no gene function being annotated for 11 down-regulated genes specific to the 6-h timepoint (Figure 4B and Supplementary Table 4).

#### 3.3.3 Differential gene expression in flutianil-treated samples

Gene expression in samples exposed to flutianil was analyzed at both 3 and 6 h, to discern any specific changes related to the impact of an EC_50_ dose of flutianil on the germination of *E. necator* conidia. At 3 h, there was up-regulation of 7 shared genes while no down-regulated genes were shared. Interestingly, maltose permease *MAL31* or MFS protein is up-regulated at 3- and 6-h time points. Unique to 3 h was up-regulated cytochrome P450 and Protein SFK1 (Suppressor of four kinase protein 1). At 6 h, MFS transporter, rifampicin monooxygenase and a chitinase were up-regulated (Figure 5A). In the down-regulated genes in flutianil treatment, there was no gene identified with an associated function (Figure 5B and Supplementary Table 4).

**Figure 5:**
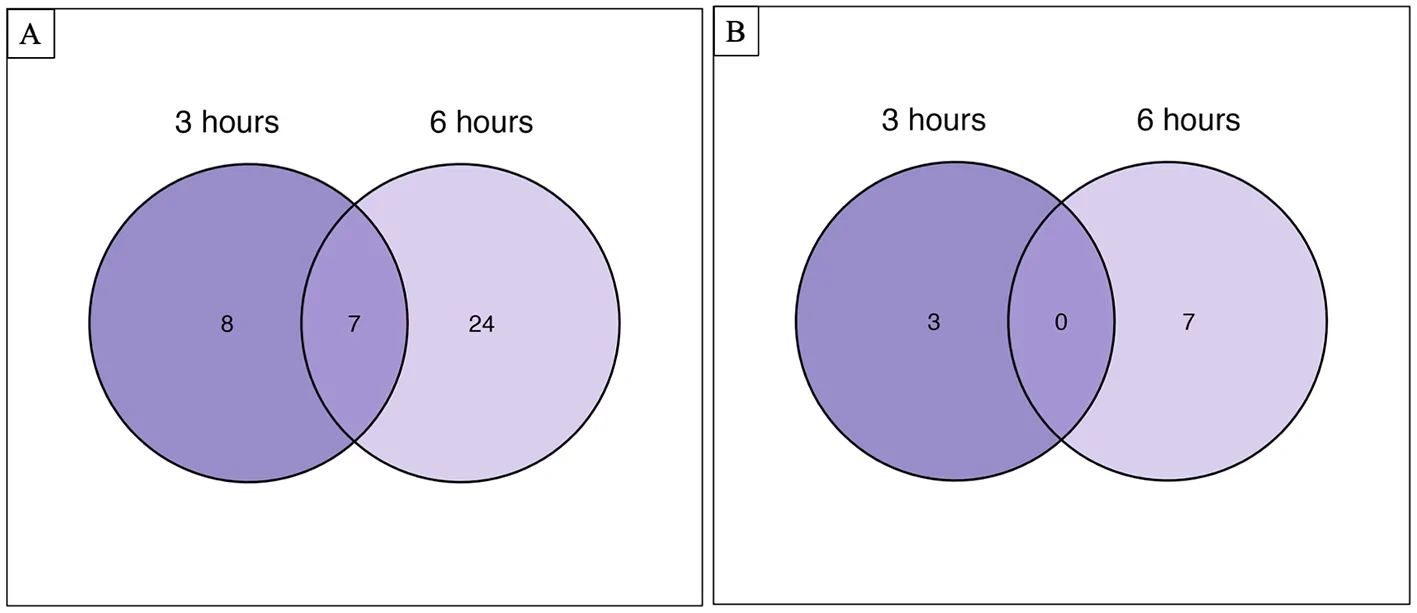
Differentially expressed genes in flutianil treatment comparing 3-h and 6-h time points. **(A)** Number of up-regulated genes, and **(B)** number of down-regulated genes.

### 3.4 Differential gene expression at 3- and 6-h durations among quinoxyfen, trifloxystrobin and flutianil treatment

#### 3.4.1 DEGs at 3-h time-point

At the 3-h time point, there were 5 common genes up-regulated in all three fungicide treatments while none was reported as a shared gene being downregulated. In the up-regulated genes, the shared genes had a Cytochrome P450 monooxygenase, with the highest log2FC of 1.54 in quinoxyfen treatment. Trifloxystrobin and quinoxyfen shared up-regulated cytochrome P450 while flutianil and trifloxystrobin shared a maltose permease *MAL31* or MFS family protein (Figure 6A).

**Figure 6:**
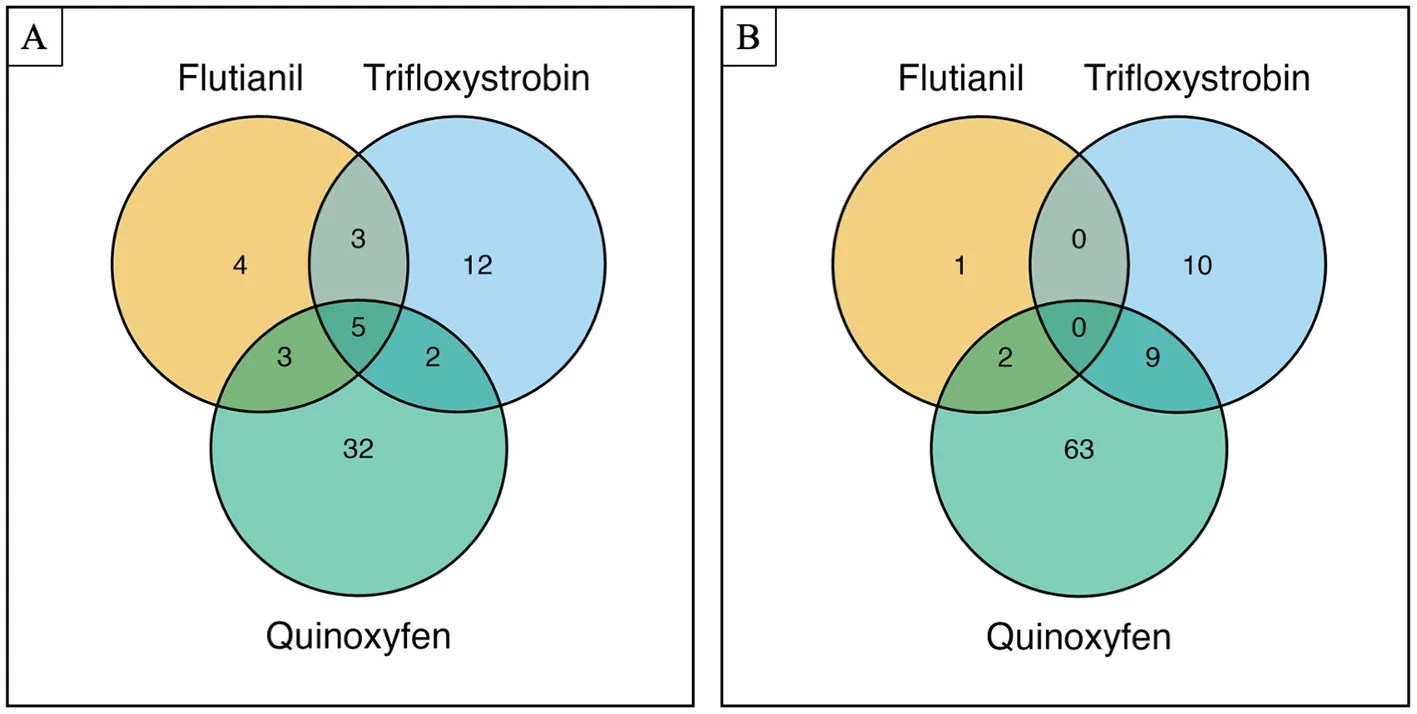
Venn diagrams show similarities and dissimilarities between the number of differentially expressed genes across treatments. Number of genes **(A)** upregulated, and **(B)** downregulated in fungicide (trifloxystrobin, flutianil, and quinoxyfen) treated *Erysiphe necator* conidia were compared to control at 3-h treatment exposure. Genes were classified as up- and down-regulated using an adjusted *p-*value of 0.05 with a log-fold change of 1.

Among the downregulated genes, at 3 h, trifloxystrobin and quinoxyfen were the only fungicides with shared genes. Among the shared downregulated genes included, Demethyl-4-deoxygadusol synthase, a chitinase and Thioredoxin-1 (Figure 6B and Table 1).

**Table 1:** Log-fold changes in commonly upregulated and downregulated genes in *Erysiphe necator* conidia treated with trifloxystrobin, quinoxyfen, and/or flutianil compared to control at 3 and 6-h exposure.

Gene ID	Gene name	Time duration (hour)	Flutianil/control	Trifloxystrobin/control	Quinoxyfen/control	Expression change
HI914_00719	Maltose permease *MAL31*	3	1.34	1.43	–	Upregulated
HI914_05519	Cytochrome P450 monooxygenase	3	1.13	1.08	1.54	Upregulated
HI914_07477	Cytochrome P450 monooxygenase aclL	3	–	1.15	1.04	Upregulated
HI914_01057	Demethyl-4-deoxygadusol synthase	3	–	−1.97	−1.70	Downregulated
HI914_03920	Thioredoxin-1	3	–	−1.27	−1.10	Downregulated
HI914_05904	Chitinase	3	–	−2.66	−2.29	Downregulated
HI914_00719	Maltose permease *MAL31*	6	1.07	1.29	1.57	Upregulated
HI914_02682	Enoyl-CoA delta isomerase 3	6	1.14	1.01	1.83	Upregulated
HI914_05871	Sphingolipid long chain base-responsive protein LSP1	6	1.08	1.16	1.67	Upregulated
HI914_06944	Fungal potassium channel	6	1.43	1.08	1.69	Upregulated
HI914_07478	Trichothecene C-4 hydroxylase	6	1.06	1.02	1.34	Upregulated
HI914_03303	Protein SFK1 (suppressor of four kinase protein 1)	6	1.02	–	1.35	Upregulated
HI914_04831	MFS transporter	6	1.48	–	1.95	Upregulated
HI914_00115	NADP-dependent oxidoreductase *RED1*	6	–	1.72	1.24	Upregulated
HI914_00677	Copper acquisition factor BIM1-like	6	–	1.52	1.84	Upregulated
HI914_02814	NADH-ubiquinone oxidoreductase	6	–	1.05	1.28	Upregulated

Control at 3 and 6-h were used as baseline to identify differentially expressed genes. Genes were classified as up- and down-regulated using an adjusted *p-*value of 0.05 with absolute log-fold change of 1.

#### 3.4.2 DEGs at 6-h time-point

At 6 h after fungicide treatment, there were 11 shared up-regulated genes while none down-regulated. Among the shared up-regulated genes in all treatments were maltose permease or MFS transporter gene and maltose permease. Flutianil and quinoxyfen shared 17 genes, among them included up-regulated MFS transporter, Protein SFK1 (Suppressor of four kinase protein 1). Further, trifloxystrobin and quinoxyfen had a shared NADP-dependent oxidoreductase *RED1* up-regulation (Figure 7A). No common down-regulated genes were reported with a known function (Figure 7B and Table 1, Supplementary Table 5).

**Figure 7:**
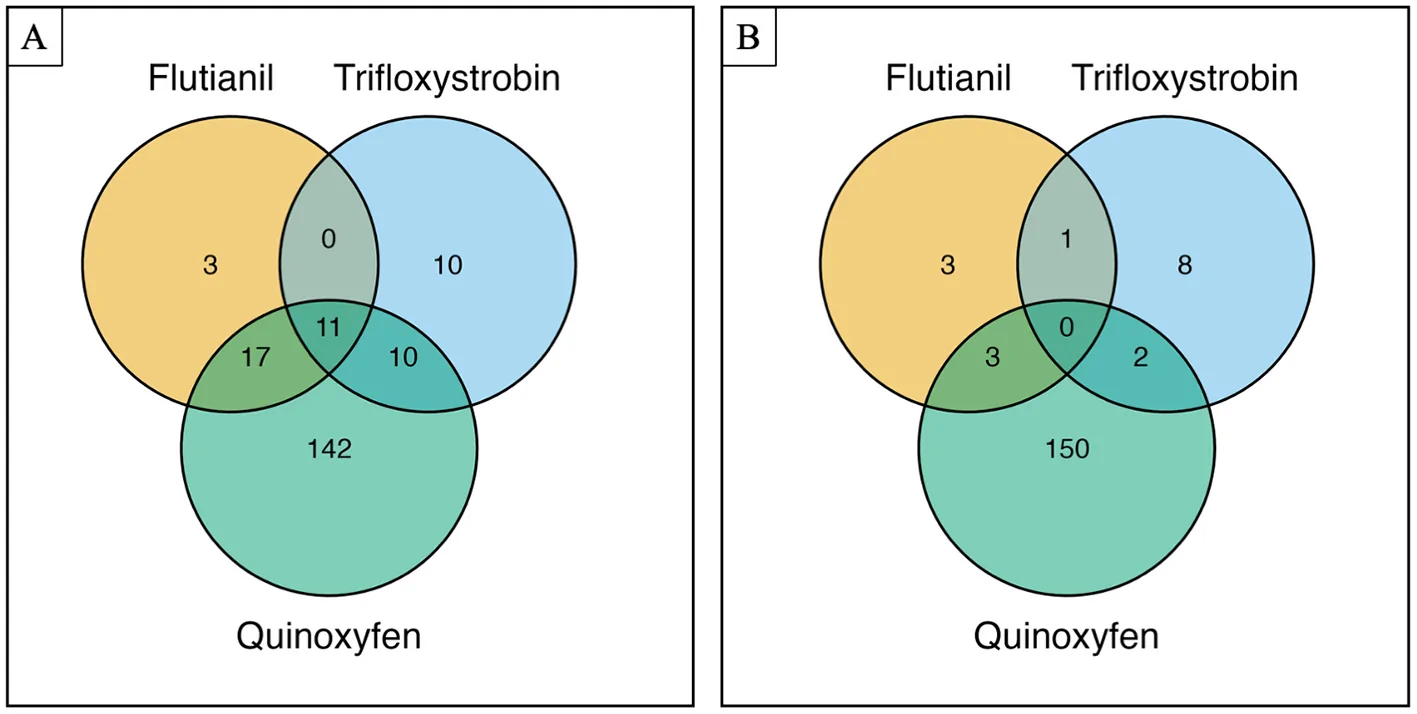
Venn diagrams show similarities and dissimilarities between the number of differentially expressed genes across treatments. Number of genes **(A)** upregulated, and **(B)** downregulated in fungicide (trifloxystrobin, flutianil, and quinoxyfen) treated *Erysiphe necator* conidia were compared to control at 6-h treatment exposure. Genes were classified as up- and down-regulated using an adjusted *p-*value of 0.05 with a log-fold change of 1.

## 4 Discussion

Quinoxyfen, metrafenone, cyflufenamid, and flutianil are among the relatively recent fungicides developed for powdery mildew (Vielba-Fernández et al., 2020). These fungicides play a crucial role in resistance management strategies for *E. necator*, especially given the widespread resistance to QoI and DMI fungicides (Miles et al., 2021; Sharma et al., 2023). An *in vitro* conidial germination test was performed in this study to establish the EC_50_ concentrations using a sensitive *E. necator* isolate for the fungicides quinoxyfen, metrafenone, cyflufenamid, flutianil and trifloxystrobin. The conidial germination of *E. necator* isolate was successfully inhibited in 50% of the conidia at determined EC_50_ concentrations of quinoxyfen, trifloxystrobin and flutianil fungicides. Cyflufenamid and metrafenone were not effective in consistently inhibiting conidial germination, suggesting that these fungicides might target different biological processes in *E. necator* than affecting conidial germination directly similar to previous research (Opalski et al., 2006; Haramoto et al., 2006). While flutianil was effective at inhibiting conidial germination, most conidia still produced germ tubes that were shorter than half the length of the conidia at 16-h duration. Our transcriptional data shows chitinase upregulation, coinciding with germ tube growth, may suggest impaired cell wall plasticity and mechanically constrain germ tube development. Thus, flutianil appears to resemble quinoxyfen and trifloxystrobin in its ability to inhibit conidial germination in *E. necator*. Since the mechanism of action for trifloxystrobin (a QoI fungicide) is well understood (Bartlett et al., 2002), it was included in the transcriptomics study to offer insights that might be compared with the effects of flutianil and quinoxyfen.

The transcriptomic study presented here provides an initial understanding of how these fungicides affect the early phase of conidial germination in *E. necator* using each fungicide's respective EC_50_ concentration. This approach serves as a valuable starting point for identifying genes that are differentially expressed at sublethal doses and offers insights into the early molecular responses and adaptations *E. necator* may undergo when exposed to chemical stress.

Our study revealed a limited overlap in the differentially expressed genes triggered by the fungicides flutianil, quinoxyfen, and trifloxystrobin, at both 3-h and 6-h intervals (Figures 6, 7). This indicates that each fungicide induces a distinct gene expression pattern in *E. necator* compared to the control, as a large number of the genes were unique to each fungicide treatment. For example, at the 6-h mark, under quinoxyfen treatment an upregulation of 190 genes was observed, with only 48 genes shared among other treatments. A similar trend was observed for downregulated genes, with few overlaps and many DEGs being specific to each fungicide treatment. These results suggest that *E. necator* responds substantially differently to flutianil, quinoxyfen, and trifloxystrobin leading to unique transcriptional responses. This points to the possibility that each fungicide targets distinct cellular or molecular pathways that disrupt different biological processes within *E. necator*.

Although the number of differentially expressed genes is limited, there is some overlap primarily in relationship to cellular respiration. At the 6-h interval, maltose permease *MAL31* (HI914_00719) was commonly upregulated across all treatments. In yeast, maltose permease facilitates maltose uptake, which is hydrolyzed into glucose and enters glycolytic pathways to fuel ATP production via the Krebs cycle and electron transport chain (Medintz et al., 1996). The upregulation of maltose permease was observed in trifloxystrobin and flutianil treatments at 3 h, and in all three fungicide treatments at 6 h, suggesting that as fungi face fungicidal stress, they increase sugar metabolism to meet elevated energy demands. Moreover, NADP-dependent oxidoreductase RED1 (HI914_00115) was upregulated at 6 h in both quinoxyfen and trifloxystrobin treatments. Also, the upregulation of enoyl-CoA delta isomerase 3 at 6 h in flutianil, trifloxystrobin and quinoxyfen treatments suggests potential involvement in fatty acid β-oxidation. Based on conserved fungal metabolic pathways, this may indicate increased lipid breakdown to generate acetyl-CoA and electron carriers (*NADH* and *FADH2*) for ATP production, reflecting a metabolic shift toward lipid utilization in response to energy demands imposed by fungicide treatment (Xin et al., 2022).

Additionally, in quinoxyfen and flutianil treatments at the 6-h interval, MFS transporters (HI914_04831) was commonly upregulated, indicating a broader fungicide induced metabolic response. The upregulation of MFS transporters suggests increased nutrient and metabolite transport involved in drug efflux systems, secretion of endogenously produced toxins, which could be necessary for sustaining cellular metabolism and detoxification processes as fungicidal exposure continues (Del Sorbo et al., 2000). This gene has also been associated with multidrug resistance in fungicides. Hence, its upregulation could potential lead to reduced sensitivity to flutianil.

Based on prior research and our findings, flutianil appears to affect multiple stages of powdery mildew fungal development, from conidial germination to haustoria formation (Kimura et al., 2020, 2021). Previous research on gene expression changes in *B. graminis* treated with flutianil during haustoria formation (Kimura et al., 2021) reported overexpression of sugar transporter and MFS transporter genes in flutianil-treated samples, which is similar to our analysis which detected upregulation of these same gene families. These findings suggest a consistent strategy in which fungi activate nutrient uptake to support energy production via glycolysis, increase membrane transport to enhance multidrug resistance and metabolite transport (potentially allowing fungi to expel toxic compounds and optimize nutrient intake) as a protective measure in response to fungicide exposure, regardless of developmental stage. In a broader context, these responses demonstrate a common adaptive mechanism in fungi, where metabolic pathways related to nutrient acquisition, cell wall modification, and efflux systems are mobilized to overcome fungicidal stress.

The transcriptional changes specifically triggered by quinoxyfen treatment suggest that it disrupts a wide array of cellular processes in the fungus, impacting metabolism, stress response, transport, signal transduction, and growth regulation. A critical component of the fungal response appears to be the regulation of signal transduction pathways, particularly those involving the MAPK (Mitogen-Activated Protein Kinase) pathway. In this study the downregulation of serine/threonine-protein kinase *STE11* was found, which is part of the MAPK. Similarly, regulation of proteins involved in the MAPK pathway has also been reported in another study conducted on quinoxyfen-treated *E. necator* isolates, along with downregulation of GTPase-mediated signal transduction. Another study has also reported genes related to GTP-binding or GTPase activity were overexpressed in quinoxyfen-treated *Blumeria graminis* f. sp. *hordei* compared to untreated samples (Wheeler et al., 2000). We also found downregulation of Ras guanine nucleotide exchange factor A (Ras-GEF A) protein in the treated *E. necator* isolate which acts as a molecular switch by cycling between a GDP-bound inactive state and a GTP-bound active state (Wittinghofer et al., 1997; Cox et al., 2014).

Moreover, the impairment of transport proteins, including glutathione transporter, Na(+)/H(+) antiporter, and aquaporins, suggests that quinoxyfen interferes with the fungus's capacity to maintain ion balance and detoxify harmful substances as these transporters are critical for redox homeostasis (Bachhawat et al., 2013). In contrast, the upregulation of genes involved in metabolic processes, such as the NADP-dependent malic enzyme and malate synthase, points to an attempt by *E. necator* to maintain energy production under stress. The upregulation of heat shock proteins, MFS and ABC multidrug transporters further underscores the activation of stress response and fungicide resistance pathways by drug efflux, indicating that quinoxyfen creates an environment that challenges protein stability and cellular integrity (Del Sorbo et al., 2000).

To build on our current findings, expanding the transcriptomic analysis to include additional time points would help capture dynamic gene expression patterns over time. Moreover, functional studies such as gene knockouts, knockdowns, RNAi or overexpression studies can help us to characterize the biological roles of these key genes. The replication of the field conditions for an obligate pathogen in the lab using *in vitro* conidial germination assay has its limitation. Further, inclusion of isolates with contrasting fungicide sensitivities can help us provide more evidence on relationship between candidate genes and resistance. Our study provides important insights into the similarities and differences in the mechanisms of key fungicides used to control powdery mildew. Among the fungicides tested: quinoxyfen, flutianil, and trifloxystrobin, only a few differentially expressed genes were shared, indicating that quinoxyfen and flutianil function through distinct mechanisms from each other and from trifloxystrobin. Upregulation of MFS transporters and ABC multidrug transporters reflects greater chances of potential risk for cross-resistance or reduced sensitivities to quinoxyfen and flutianil. These findings can guide the development of more effective strategies for fungicide resistance management. This can help in extending the effectiveness of these fungicides for longer durations in controlling grape powdery mildew.

## Data Availability

The raw RNA-seq datasets generated and analyzed in this study have been deposited in the NCBI BioProject repository under accession number PRJNA1474447. The scripts used can be found at Github repo https://github.com/mohitmahey/RNA_seq_analysis.

## Supplementary material

Supplementary Figure 1 Percentage of conidial germination of *Erysiphe necator* relative to the control after treatment with fungicides at different concentrations. Conidial germination assay was conducted using a sensitive isolate of *E. necator*. Each concentration had three technical replicates and the experiment was repeated twice. Germination assay treated with **(A)** metrafenone, **(B)** cyflufenamid. The point represent average relative germination (%) while error bars represent standard error.

Supplementary Figure 2 PCA analysis of the samples **(A)** before batch correction, **(B)** after batch correction using combat-seq.

Supplementary Table 1 Quality control metrics of the various samples used in this study.

Supplementary Table 2 Log2-fold changes in upregulated and downregulated genes in Erysiphe necator conidia treated with quinoxyfen compared to control at 3-h exposure. Genes with unknown function were removed. Genes were classified as up- and down-regulated using an adjusted *p*-value of 0.05 with absolute log2-fold change of 1.

Supplementary Table 3 Log2-fold changes in upregulated and downregulated genes in Erysiphe necator conidia treated with quinoxyfen compared to control at 6-h exposure. Genes with unknown function were removed. Genes were classified as up- and down-regulated using an adjusted *p*-value of 0.05 with absolute log-fold change of 1.

Supplementary Table 4 Log2-fold changes in uniquely upregulated and downregulated genes in Erysiphe necator conidia treated with trifloxystrobin or flutianil. Control at 3 and 6-h were used as baseline to identify differentially expressed genes. Genes were classified as up- and down-regulated using an adjusted *p*-value of 0.05 with absolute log-fold change of 1.

Supplementary Table 5 Unknown gene functions having common downregulation at 6 the hour time-point among various treatments.
