# Supplementary material for: Sub-lethal dose exposure of cyflufenamid, flutianil, quinoxyfen, trifloxystrobin and metrafenone effects on conidial germination and gene expression in the grape powdery mildew pathogen
Source: Front Microbiol. 2026 Oct 12;17:1944688. doi: 10.3389/fmicb.2026.1944688 (PMC13696460; doi:10.3389/fmicb.2026.1944688)
Supplement: Supplementary file 1 [file Table_1.docx]

Supplementary Table 1: Quality control metrics of the various samples used in this study.

| Sample ID | Treatment | Time points | Batch | Duplication rate (%) | Percentage mapped uniquely (%) | Multimapped reads (%) |
| --- | --- | --- | --- | --- | --- | --- |
| C02 | Control | 0 hours | 2 | 34.00 | 87.11 | 9.21 |
| C03 | Control | 0 hours | 3 | 35.10 | 87.14 | 9.18 |
| C31 | Control | 3 hours | 4 | 35.30 | 91.20 | 4.72 |
| C32 | Control | 3 hours | 2 | 36.50 | 89.98 | 6.57 |
| C62 | Control | 6 hours | 2 | 36.10 | 87.85 | 8.46 |
| C63 | Control | 6 hours | 3 | 35.50 | 85.67 | 9.08 |
| G31 | Flutianil | 3 hours | 1 | 36.40 | 89.82 | 7.11 |
| G32 | Flutianil | 3 hours | 2 | 38.80 | 86.22 | 10.22 |
| G61 | Flutianil | 6 hours | 1 | 37.50 | 90.83 | 4.79 |
| G63 | Flutianil | 6 hours | 3 | 39.30 | 80.48 | 11.36 |
| Q31 | Quinoxyfen | 3 hours | 1 | 35.10 | 86.52 | 10.00 |
| Q32 | Quinoxyfen | 3 hours | 4 | 36.00 | 91.31 | 5.06 |
| Q33 | Quinoxyfen | 3 hours | 3 | 34.20 | 89.52 | 5.87 |
| Q62 | Quinoxyfen | 6 hours | 2 | 35.20 | 86.61 | 9.20 |
| Q63 | Quinoxyfen | 6 hours | 3 | 39.10 | 79.94 | 11.15 |
| F31 | Trifloxystrobin | 3 hours | 1 | 35.10 | 88.06 | 8.35 |
| F32 | Trifloxystrobin | 3 hours | 4 | 39.00 | 87.36 | 8.96 |
| F61 | Trifloxystrobin | 6 hours | 1 | 35.60 | 85.88 | 10.10 |
| F62 | Trifloxystrobin | 6 hours | 2 | 38.60 | 86.99 | 8.67 |

Supplementary Table 2: Log2-fold changes in upregulated and downregulated genes in *Erysiphe necator* conidia treated with quinoxyfen compared to control at 3-hour exposure. Genes with unknown function were removed. Genes were classified as up- and down-regulated using an adjusted *p-value* of 0.05 with absolute log2-fold change of 1.

| Gene ID | Gene Function | Log2-fold Change | Expression Change |
| --- | --- | --- | --- |
| HI914_05519 | Cytochrome P450 monooxygenase | 1.54 | Upregulation |
| HI914_04859 | Velvet complex subunit 2 | 1.34 | Upregulation |
| HI914_03303 | Protein SFK1 | 1.31 | Upregulation |
| HI914_01636 | Oleate hydroxylase FAH12 | 1.30 | Upregulation |
| HI914_00981 | Secreted RxLR effector protein | 1.27 | Upregulation |
| HI914_06764 | Spore development regulator RYP2 | 1.25 | Upregulation |
| HI914_01388 | Diphosphoinositol polyphosphate phosphohydrolase aps1 | 1.13 | Upregulation |
| HI914_03392 | putative yippee family protein | 1.08 | Upregulation |
| HI914_05073 | putative inorganic phosphate transporter 1-6 | 1.07 | Upregulation |
| HI914_03138 | putative sucrose utilization protein | 1.05 | Upregulation |
| HI914_07477 | Cytochrome P450 61 | 1.04 | Upregulation |
| HI914_03688 | thiamine pyrophosphokinase | -1.01 | Downregulation |
| HI914_05032 | Nicotinate catabolism cluster-specific transcription factor | -1.01 | Downregulation |
| HI914_04931 | HMG box-containing protein | -1.03 | Downregulation |
| HI914_02059 | Hexokinase-1 | -1.05 | Downregulation |
| HI914_02426 | Serine/threonine-protein kinase mph1 | -1.06 | Downregulation |
| HI914_06808 | Cholinesterase | -1.07 | Downregulation |
| HI914_07444 | Orotate phosphoribosyltransferase | -1.08 | Downregulation |
| HI914_02516 | Pyrroline-5-carboxylate reductase | -1.08 | Downregulation |
| HI914_00994 | Acetylesterase | -1.16 | Downregulation |
| HI914_03305 | Catabolic L-serine/threonine dehydratase | -1.18 | Downregulation |
| HI914_00921 | O-acetyltransferase PaAT-1 | -1.25 | Downregulation |
| HI914_00897 | Global transcription regulator sge1 | -1.26 | Downregulation |
| HI914_05967 | G2/mitotic-specific cyclin-4 | -1.34 | Downregulation |
| HI914_04132 | Plasma membrane ATPase | -1.34 | Downregulation |
| HI914_03453 | putative rare lipoprotein a -like double-psi beta-barrel domain-containing | -1.40 | Downregulation |
| HI914_00736 | Transcription factor vib-1 | -1.42 | Downregulation |
| HI914_02418 | putative extracellular matrix protein | -1.46 | Downregulation |
| HI914_00681 | Cell wall mannoprotein CIS3 | -1.49 | Downregulation |
| HI914_05416 | Glucan 12C3-beta-glucosidase | -1.55 | Downregulation |
| HI914_03875 | putative endo-beta-12C4-glucanase D | -1.59 | Downregulation |
| HI914_01057 | Demethyl-4-deoxygadusol synthase | -1.70 | Downregulation |
| HI914_05943 | Catalase-peroxidase | -1.76 | Downregulation |
| HI914_05905 | Gamma-glutamylputrescine oxidoreductase | -1.77 | Downregulation |
| HI914_05904 | Chitinase | -2.29 | Downregulation |
| HI914_07067 | Agglutinin | -4.48 | Downregulation |

Supplementary Table 3: Log2-fold changes in upregulated and downregulated genes in *Erysiphe necator* conidia treated with quinoxyfen compared to control at 6-hour exposure. Genes with unknown function were removed. Genes were classified as up- and down-regulated using an adjusted p-*value* of 0.05 with absolute log-fold change of 1

| Gene ID | Log2-fold Change | Gene Function | Expression Change |
| --- | --- | --- | --- |
| HI914_04831 | 1.95 | MFS transporter | Upregulation |
| HI914_04743 | 1.84 | heat shock protein | Upregulation |
| HI914_02682 | 1.83 | Enoyl-CoA delta isomerase 3 | Upregulation |
| HI914_03314 | 1.80 | Cutinase | Upregulation |
| HI914_03968 | 1.70 | heat shock protein | Upregulation |
| HI914_06944 | 1.69 | Fungal potassium channel | Upregulation |
| HI914_05871 | 1.67 | Sphingolipid long chain base-responsive protein LSP1 | Upregulation |
| HI914_05697 | 1.64 | ABC multidrug transporter | Upregulation |
| HI914_03415 | 1.62 | Clock-controlled protein | Upregulation |
| HI914_04741 | 1.58 | heat shock protein | Upregulation |
| HI914_00719 | 1.57 | Maltose permease MAL31 | Upregulation |
| HI914_00178 | 1.54 | Cytochrome P450 | Upregulation |
| HI914_02452 | 1.52 | Rifampicin monooxygenase | Upregulation |
| HI914_04859 | 1.49 | Velvet complex subunit 2 | Upregulation |
| HI914_04746 | 1.48 | heat shock protein | Upregulation |
| HI914_01636 | 1.47 | Oleate hydroxylase FAH12 | Upregulation |
| HI914_04217 | 1.39 | heat shock protein | Upregulation |
| HI914_01388 | 1.34 | Diphosphoinositol polyphosphate phosphohydrolase aps1 | Upregulation |
| HI914_07478 | 1.34 | Trichothecene C-4 hydroxylase | Upregulation |
| HI914_04980 | 1.33 | NADP-dependent malic enzyme | Upregulation |
| HI914_05519 | 1.31 | Cytochrome P450 monooxygenase | Upregulation |
| HI914_00716 | 1.30 | Glucose-repressible protein | Upregulation |
| HI914_02814 | 1.28 | NADH-ubiquinone oxidoreductase | Upregulation |
| HI914_04568 | 1.25 | Secondary metabolism regulator LAE1 | Upregulation |
| HI914_06032 | 1.25 | Eukaryotic translation initiation factor 4E type 3 | Upregulation |
| HI914_00115 | 1.24 | NADP-dependent oxidoreductase RED1 | Upregulation |
| HI914_02627 | 1.22 | 3-oxoacyl-[acyl-carrier-protein] reductase FabG | Upregulation |
| HI914_03811 | 1.21 | Queuine tRNA-ribosyltransferase catalytic subunit | Upregulation |
| HI914_02641 | 1.21 | Malate synthase2C glyoxysomal | Upregulation |
| HI914_04838 | 1.20 | Haemolysin-III related | Upregulation |
| HI914_06857 | 1.19 | Grainyhead-like protein | Upregulation |
| HI914_07477 | 1.18 | Cytochrome P450 monooxygenase aclL | Upregulation |
| HI914_02873 | 1.18 | Mannan endo-12C6-alpha-mannosidase DCW1 | Upregulation |
| HI914_05131 | 1.14 | Mechanosensitive ion channel protein Msy1 | Upregulation |
| HI914_01640 | 1.14 | putative secreted beta-glucosidase | Upregulation |
| HI914_00322 | 1.14 | Glucan 12C3-beta-glucosidase | Upregulation |
| HI914_00838 | 1.11 | Enhancer of polycomb-like protein 1 | Upregulation |
| HI914_02337 | 1.10 | Iron transport multicopper oxidase fetC | Upregulation |
| HI914_02561 | 1.10 | Very-long-chain 3-oxoacyl-CoA reductase | Upregulation |
| HI914_01146 | 1.09 | General transcription and DNA repair factor IIH subunit TFB5 | Upregulation |
| HI914_04464 | 1.09 | Peroxisomal membrane associated protein 20 | Upregulation |
| HI914_03302 | 1.09 | Serine/threonine-protein kinase KIN28 | Upregulation |
| HI914_03067 | 1.08 | Pathogenesis associated protein Cap20 | Upregulation |
| HI914_04413 | 1.05 | Ras-related protein Rab-18 | Upregulation |
| HI914_01967 | 1.05 | Thioredoxin-1 | Upregulation |
| HI914_07162 | 1.04 | Catechol dioxygenase | Upregulation |
| HI914_01691 | 1.03 | heat shock protein | Upregulation |
| HI914_02858 | 1.03 | FAD-dependent monooxygenase asL6 | Upregulation |
| HI914_02466 | 1.02 | heat shock protein | Upregulation |
| HI914_03112 | 1.01 | PRKR-interacting protein 1 | Upregulation |
| HI914_07317 | 1.01 | Copper resistance protein CRF1 | Upregulation |
| HI914_05760 | 1.01 | Cytochrome c oxidase assembly protein COX19 | Upregulation |
| HI914_07101 | 1.00 | Guanine nucleotide-binding protein alpha-3 subunit | Upregulation |
| HI914_03499 | 1.00 | Protein N-terminal amidase | Upregulation |
| HI914_00323 | 1.00 | IgA FC receptor | Upregulation |
| HI914_04557 | -1.00 | Amidophosphoribosyltransferase | Downregulation |
| HI914_00722 | -1.01 | putative metallophosphoesterase | Downregulation |
| HI914_03322 | -1.01 | CHITIN SYNTHASE 3 COMPLEX PROTEIN | Downregulation |
| HI914_02394 | -1.01 | Chitin synthase export chaperone | Downregulation |
| HI914_02488 | -1.01 | putative peptide transporter ptr2 | Downregulation |
| HI914_01218 | -1.02 | Biofilm regulator 1 | Downregulation |
| HI914_02487 | -1.02 | putative peptide transporter ptr2 | Downregulation |
| HI914_00117 | -1.03 | IQ domain-containing protein IQM6 | Downregulation |
| HI914_02252 | -1.04 | Low affinity vacuolar monovalent cation/H(+) antiporter | Downregulation |
| HI914_02048 | -1.05 | Receptor expression-enhancing protein 2 | Downregulation |
| HI914_07225 | -1.05 | DNA topoisomerase 2 | Downregulation |
| HI914_06863 | -1.05 | Iron-sulfur clusters transporter atm12C mitochondrial | Downregulation |
| HI914_00854 | -1.08 | Inner kinetochore subunit mis6 | Downregulation |
| HI914_07287 | -1.09 | Peptidase S41 family protein ustP | Downregulation |
| HI914_01895 | -1.10 | Tyrosine-protein phosphatase cdcA | Downregulation |
| HI914_00620 | -1.14 | Sexual differentiation process protein isp4 | Downregulation |
| HI914_02608 | -1.14 | Serine/threonine-protein kinase sid2 | Downregulation |
| HI914_01231 | -1.16 | DNA replication licensing factor mcm2 | Downregulation |
| HI914_03093 | -1.17 | Vesicular-fusion protein sec18 | Downregulation |
| HI914_04670 | -1.18 | Histone H4 | Downregulation |
| HI914_03141 | -1.19 | Endothelin-converting enzyme | Downregulation |
| HI914_05663 | -1.19 | DNA polymerase lambda | Downregulation |
| HI914_04032 | -1.20 | DNA polymerase zeta processivity subunit | Downregulation |
| HI914_00580 | -1.20 | Transcriptional activator hac1 | Downregulation |
| HI914_00845 | -1.21 | Serine/threonine-protein kinase STE11 | Downregulation |
| HI914_00053 | -1.21 | Carboxypeptidase S1 | Downregulation |
| HI914_03671 | -1.23 | Ingression protein fic1 | Downregulation |
| HI914_01676 | -1.24 | Transcriptional regulatory protein pro1 | Downregulation |
| HI914_03735 | -1.24 | Ras guanine nucleotide exchange factor A | Downregulation |
| HI914_02422 | -1.25 | Chanoclavine-I aldehyde reductase fgaOx3 | Downregulation |
| HI914_04354 | -1.25 | Major Facilitator Superfamily | Downregulation |
| HI914_03792 | -1.25 | Vacuolar calcium ion transporter | Downregulation |
| HI914_03453 | -1.28 | Allergen Asp F7 | Downregulation |
| HI914_05102 | -1.29 | Serine/threonine-protein kinase ark1 | Downregulation |
| HI914_07433 | -1.30 | putative cardiolipin-specific deacylase 12C mitochondrial | Downregulation |
| HI914_06405 | -1.31 | Bud site selection protein BUD4 | Downregulation |
| HI914_01062 | -1.31 | Amino-acid permease | Downregulation |
| HI914_02088 | -1.34 | Kinesin-like motor protein 9 | Downregulation |
| HI914_05438 | -1.35 | Carboxypeptidase Y A | Downregulation |
| HI914_03895 | -1.35 | Chitin synthase 2 | Downregulation |
| HI914_02876 | -1.37 | G2-specific protein kinase nim-1 | Downregulation |
| HI914_06808 | -1.38 | Cholinesterase | Downregulation |
| HI914_03419 | -1.38 | Major facilitator-type transporter ecdD | Downregulation |
| HI914_04140 | -1.40 | Glutathione transporter | Downregulation |
| HI914_04132 | -1.41 | Plasma membrane ATPase | Downregulation |
| HI914_01891 | -1.42 | Cell surface Cu-only superoxide dismutase | Downregulation |
| HI914_01840 | -1.42 | G2/mitotic-specific cyclin-B | Downregulation |
| HI914_01589 | -1.42 | Glycosyl hydrolases family 16 | Downregulation |
| HI914_00921 | -1.45 | O-acetyltransferase PaAT-1 | Downregulation |
| HI914_00205 | -1.46 | Vacuolar calcium ion transporter | Downregulation |
| HI914_02516 | -1.48 | Pyrroline-5-carboxylate reductase | Downregulation |
| HI914_06898 | -1.51 | Metallothionein expression activator | Downregulation |
| HI914_06615 | -1.55 | putative Na(+)/H(+) antiporter | Downregulation |
| HI914_04441 | -1.56 | Homoserine kinase | Downregulation |
| HI914_03910 | -1.57 | Aquaporin | Downregulation |
| HI914_07567 | -1.61 | Aspercryptin biosynthesis cluster-specific transcription regulator atnN | Downregulation |
| HI914_04931 | -1.69 | HMG box-containing protein | Downregulation |
| HI914_03875 | -1.74 | putative endo-beta-12C4-glucanase D | Downregulation |
| HI914_05905 | -1.76 | Gamma-glutamylputrescine oxidoreductase | Downregulation |
| HI914_05239 | -1.77 | Pleckstrin homology domain | Downregulation |
| HI914_07444 | -1.81 | Orotate phosphoribosyltransferase | Downregulation |
| HI914_06070 | -1.86 | Anaphase spindle elongation protein 1 | Downregulation |
| HI914_02426 | -1.90 | Serine/threonine-protein kinase mph1 | Downregulation |
| HI914_05967 | -1.97 | G2/mitotic-specific cyclin-4 | Downregulation |
| HI914_00736 | -1.97 | Transcription factor vib-1 | Downregulation |
| HI914_06793 | -2.19 | putative GPI-anchored cupredoxin | Downregulation |
| HI914_00897 | -2.19 | Global transcription regulator sge1 | Downregulation |
| HI914_03305 | -2.22 | Catabolic L-serine/threonine dehydratase | Downregulation |
| HI914_06111 | -2.27 | Beta-12C2-xylosyltransferase 1 | Downregulation |
| HI914_02716 | -2.28 | Glycosyl hydrolase family 92 | Downregulation |
| HI914_05943 | -2.29 | Catalase-peroxidase | Downregulation |
| HI914_00240 | -2.35 | WD repeat-containing protein slp1 | Downregulation |
| HI914_07584 | -2.36 | ATP-dependent RNA helicase dbp-4 | Downregulation |
| HI914_05416 | -2.58 | Glucan 12C3-beta-glucosidase | Downregulation |
| HI914_06303 | -2.70 | Aquaporin-3 | Downregulation |
| HI914_04388 | -2.79 | Oxalate--CoA ligase | Downregulation |
| HI914_01795 | -2.80 | putative gpi anchored serine-threonine rich protein | Downregulation |
| HI914_06607 | -2.99 | Ribonucleoside-diphosphate reductase small chain | Downregulation |
| HI914_02770 | -3.76 | Galactitol 2-dehydrogenase | Downregulation |

Supplementary Table 4: Log2-fold changes in uniquely upregulated and downregulated genes in *Erysiphe necator* conidia treated with trifloxystrobin or flutianil. Control at 3 and 6-hours were used as baseline to identify differentially expressed genes. Genes were classified as up- and down-regulated using an adjusted *p-value* of 0.05 with absolute log-fold change of 1.

| Gene ID | Gene Name | Time Duration (hour) | Trifloxystrobin/ Control | Flutianil/ Control | Expression Change |
| --- | --- | --- | --- | --- | --- |
| HI914_03303 | Frag1/DRAM/Sfk1 family | 3 | 1.00 | - | Upregulated |
| HI914_04252 | Zinc finger CCHC-type profile. | 3 | 1.24 | - | Upregulated |
| HI914_04326 | putative peroxygenase | 3 | 1.02 | - | Upregulated |
| HI914_05519 | Cytochrome P450 monooxygenase | 3 | 1.08 | - | Upregulated |
| HI914_07477 | Cytochrome P450 monooxygenase aclL | 3 | 1.15 | - | Upregulated |
| HI914_00232 | Appressoria-specific virulence factor GAS | 3 | -1.01 | - | Downregulated |
| HI914_00987 | O-methyltransferase VdtC | 3 | -1.10 | - | Downregulated |
| HI914_01057 | Demethyl-4-deoxygadusol synthase | 3 | -1.97 | - | Downregulated |
| HI914_02615 | Plasma membrane proteolipid 3 | 3 | -1.26 | - | Downregulated |
| HI914_03920 | Thioredoxin-1 | 3 | -1.27 | - | Downregulated |
| HI914_05456 | Vacuolar membrane amino acid uptake transporter fnx2 | 3 | -1.30 | - | Downregulated |
| HI914_05904 | Chitinase | 3 | -2.66 | - | Downregulated |
| HI914_05987 | Potassium transport protein 1 | 3 | -1.35 | - | Downregulated |
| HI914_06075 | Sporulation-specific protein 13 | 3 | -1.73 | - | Downregulated |
| HI914_07382 | Mitochondrial glycine transporter | 3 | -1.02 | - | Downregulated |
| HI914_05519 | Cytochrome P450 monooxygenase | 3 | - | 1.13 | Upregulated |
| HI914_00677 | Copper acquisition factor BIM1-like | 6 | 1.52 | - | Upregulated |
| HI914_02682 | Enoyl-CoA delta isomerase 3 | 6 | 1.01 | - | Upregulated |
| HI914_02814 | NADH-ubiquinone oxidoreductase | 6 | 1.05 | - | Upregulated |
| HI914_06944 | Fungal potassium channel | 6 | 1.08 | - | Upregulated |
| HI914_02682 | Enoyl-CoA delta isomerase | 6 | - | 1.14 | Upregulated |
| HI914_04831 | MFS transporter | 6 | - | 1.48 | Upregulated |
| HI914_05871 | Sphingolipid long chain base-responsive protein LSP1 | 6 | - | 1.08 | Upregulated |
| HI914_06944 | Fungal potassium channel | 6 | - | 1.43 | Upregulated |
| HI914_07478 | Cytochrome P450 | 6 | - | 1.06 | Upregulated |

Supplementary Table 5: Unknown gene functions having common downregulation at 6 the hour time-point among various treatments.

| Gene IDs | Treatments | Log2FC Flutianil | Log2FC Trifloxystrobin | Log2FC Quinoxyfen |
| --- | --- | --- | --- | --- |
| HI914_02415 | Trifloxystrobin and Quinoxyfen | - | -1.29 | -1.43 |
